# Evidence of maternal transfer of antigen-specific antibodies in serum and breast milk to infants at high-risk of *S. pneumoniae* and *H. influenzae* disease

**DOI:** 10.3389/fimmu.2022.1005344

**Published:** 2022-09-21

**Authors:** Kelly M. Martinovich, Elke J. Seppanen, Amy S. Bleakley, Sharon L. Clark, Ross M. Andrews, Peter C. Richmond, Michael J. Binks, Ruth B. Thornton, Lea-Ann S. Kirkham

**Affiliations:** ^1^ Wesfarmers Centre of Vaccines and Infectious Diseases, Telethon Kids Institute, Perth, WA, Australia; ^2^ Menzies School of Health Research Charles, Darwin University, Darwin, NT, Australia; ^3^ School of Medicine, University of Western Australia, Perth, WA, Australia; ^4^ Research School of Population Health, Australian National University, Canberra, ACT, Australia; ^5^ Centre for Child Health Research, University of Western Australia, Perth, WA, Australia

**Keywords:** *Haemophilus influenzae*, *Streptococcus pneumoniae*, maternal, breast milk, IgG, IgA

## Abstract

**Introduction:**

Children in low-mid income countries, and First Nations children in high-income countries, experience disproportionately high rates of *Streptococcus pneumoniae* and *Haemophilus influenzae* infections and diseases including pneumonia and otitis media. We previously observed that infants from Papua New Guinea had no evidence of waning maternal immunity for *H. influenzae*-specific antibodies. In this study, we assessed *S. pneumoniae* and *H. influenzae* antibody titres in Australian First Nation mothers and infants to determine antigen-specific antibody ontogenies and whether *H. influenzae* antibody titres in infants were due to low maternal antibody titres or lack of placental transfer.

**Methods:**

Breast milk, infant nasopharyngeal swabs and ear assessment data were collected 1-, 2-, 7-months post-birth as well as maternal, cord and 7-month-old infant sera, from 85 Australian Aboriginal and Torres Strait Islander mother-infant pairs. Serum IgG and breast milk IgG and IgA antibody titres to *S. pneumoniae* antigens (PspA1, PspA2, CbpA, Ply) and *H. influenzae* antigens (PD, ChimV4, OMP26, rsPilA) were measured.

**Results:**

IgG titres in maternal and cord sera were similar for all antigens, except Ply (higher in cord; p=0.004). Sera IgG titres at 7-months of age were lower than cord sera IgG titres for all *S. pneumoniae* antigens (p<0.001). Infant sera IgG titres were higher than cord sera for *H. influenzae* PD (p=0.029), similar for OMP26 (p=0.817) and rsPilA (p=0.290), and lower for ChimV4 (p=0.004). Breast milk titres were similar for all antigens at 1, 2 and 7-months except OMP26 IgA (lower at 7-months than 1-month; p=0.035), PspA2 IgG (p=0.012) and Ply IgG that increased by 7-months (p=0.032). One third of infants carried nontypeable *Haemophilus influenzae* (NTHi), 45% carried *S. pneumoniae and* 52% had otitis media (OM) observed at least once over the 7-months. 73% of infants who carried either *S. pneumoniae or* NTHi, also had otitis media observed.

**Conclusions:**

Similarities between maternal and cord IgG titres, and absence of waning, support a lack of maternal *H. influenzae* IgG antibodies available for cross-placental transfer. Increased maternal anti-PD IgG could offer some protection from early carriage with NTHi, and maternal immunisation strategies should be considered for passive-active immunisation of infants to protect against *S. pneumoniae* and *H. influenzae* diseases.

**Trial registration:**

ClinicalTrials.gov NCT00714064 and NCT00310349.

## Introduction

Early life protection from infection is generally provided by the trans-placental transfer of IgG antibodies from mother to infant. These antibodies circulate in the infant’s bloodstream and decline over the first 6- to 12-months of life (1, 2). Passive transfer of maternal IgA antibodies through colostrum and breast milk also occurs when the child is breastfed. These antibodies are supplied for the duration of breastfeeding and protect against infections of the gastrointestinal and respiratory tracts (3, 4). The provision of maternal antibodies offers valuable protection to infants too young to be vaccinated or where vaccines are not yet available. Several maternal vaccines are available or in development, offering protection for infants against respiratory pathogens including influenza (5), pertussis (6), respiratory syncytial virus (RSV) (7), and SARS-CoV-2 (8).

*Streptococcus pneumoniae* and *Haemophilus influenzae* infections cause otitis media (OM), pneumonia and bacteraemia, which are major contributors to childhood mortality and morbidity, despite the widespread use of pneumococcal conjugate vaccines (PCVs) and the *H influenzae* type b (Hib) vaccine (9). The burden of *S. pneumoniae* and *H. influenzae* disease is particularly high in infants (10), the elderly (11, 12), in populations of low-middle-income countries, and for First Nation people in high-income countries (13). Unfortunately, current vaccines offer protection against a limited number of *S. pneumoniae* serotypes (14), and there is no licensed vaccine for nontypeable *H. influenzae* (NTHi), which is now the leading cause of *H. influenzae* infections (15). Thus, serotype-independent vaccines are being developed including whole-cell and protein-based subunit-vaccines for *S. pneumoniae* (16) and *H. influenzae* (17, 18, 19, 20) in attempt to offer broader protection against these bacterial pathogens.

Measuring the concentrations of circulating antibodies against putative vaccine candidate antigens following natural pathogen exposure provides a valuable indication of the antigen immunogenicity and in terms of maternal vaccines, the transplacental antigen-specific antibody transfer efficiency. We previously observed the natural waning of sera IgG to *S. pneumoniae*, but not *H. influenzae*, protein antigens between birth and 9 months of age among Papua New Guinean (PNG) infants (21). However, it was unclear due to lack of appropriately matched samples whether the lack of observed waning simply related to low baseline levels due to either (i) low maternal levels or (ii) poor transplacental transfer.

Antibody waning to *S. pneumoniae* antigens has also been shown in Finnish, Filipino, and North American children (22, 23, 24). Efficient cross-placental transfer of *S. pneumoniae* anti-Ply and anti-PspA antibodies has been observed in a PNG study (25). Maternal transfer of *H. influenzae* specific antibodies, to our knowledge, has not been investigated before. This is critical to understanding the waning dynamics. A cross-sectional study in China revealed that antibody titres to *H. influenzae* protein antigens Protein D (PD) and Protein 6 are at their lowest in adults of childbearing age (26), suggesting that the lack of *H. influenzae* specific antibody waning in infants may be due to low maternal titres available for placental transfer.

Understanding maternal-infant antibody titres to vaccine candidate antigens will help provide information for vaccine strategies for the development of future *S. pneumoniae* and *H. influenzae* vaccines. For example, increasing maternal titres of protective antibody through maternal immunisation may help provide enhanced protection against *S. pneumoniae* and *H. influenzae* infections from birth. While there is insufficient evidence on the efficacy of maternal pneumococcal polysaccharide vaccines providing protection against *S. pneumoniae* carriage and disease in infants (27, 28), protein-based vaccines such as pertussis are now routinely administered during pregnancy to protect infants in the first months of life (6). Thus, it is possible that future maternal immunisation strategies for protein-based *S. pneumoniae* and *H. influenzae* vaccines are viable options.

The level of maternal antibody waning in an infant is dependent on the initial maternal transfer titre. It is important to understand maternal transfer and subsequent waning of maternal antibodies when considering maternal vaccination strategies (29). To investigate further the maternal transfer of naturally induced antibody to *S. pneumoniae* antigens and *H. influenzae* antigens, antigen-specific IgG titres were compared between maternal and cord blood sera collected at delivery, and infant sera collected at 7-months of age, from Australian Aboriginal and Torres Strait Islander families. Antigen-specific IgA and IgG titres were also measured in breast milk samples collected from the mothers at 1-, 2- and 7-months post-delivery. Here we aimed to understand the potential protection afforded by systemic and mucosal protein specific antibody against colonisation and disease, the relationships between antibody titres, and nasopharyngeal colonisation, and the development of OM in the first 7-months. While these vaccine candidates are still in early clinical development, it is important to understand whether antibody to these candidate antigens can be passed from mother to infant.

## Methods

### Study cohort

This was a retrospective analysis of samples and clinical data collected from Aboriginal and Torres Strait Islander women and infants in the Northern Territory during the PneuMum randomized controlled trial (2006-2011) (30). Briefly, women in the PneuMum study were randomized to receive the 23-valent pneumococcal polysaccharide vaccine (23vPPV) 1) either while pregnant, 2) within 72 hours of delivering their baby, or 3) at the end of the study (offered only). All infants were offered their routine PCV as recommended at 2-, 4- and 6-months of age (7vPCV from June 2001 or 10vPHiD-CV from October 2009) (30). By the 7-month visit, 72% of infants had received 2 PCV doses and 52% had received 3 doses. Two infants providing samples for this antibody study received 10vPHiD-CV, which contains PD and were thus excluded from the sera IgG PD analysis.

### Ethics approval and community involvement

Approval was received from the Human Research Ethics Committee of the Northern Territory Department of Health and Menzies School of Health Research HREC and the Aboriginal Ethics Sub-Committee (HREC 2020-3800). Extensive community consultation with the PneuMum Indigenous Reference Group in the Northern Territory, the Menzies Child Health Division First Nations Reference group, the Kulunga Aboriginal Unit at Telethon Kids Institute, and the Ear Health Aboriginal Community Advisory Group in Western Australia ensured culturally appropriate processes were maintained for the additional analysis of collected specimens.

### Sample collection and processing

Maternal venous blood and cord blood samples were collected at birth, and infant blood samples were collected at 7-months of age (30). Sera was separated from blood and stored at -70°C. Manually expressed breast milk was collected at 1-, 2- and 7-months post-birth and transported at 4°C to the laboratory and centrifuged at 10,000 *g* for 30 minutes at 4°C to remove fat, with supernatant stored at -70°C until required. Nasopharyngeal swabs (NPS) were collected from the mother on the day of birth, and from the infant at 1-, 2- and 7-months of age. Swabs were placed in skim milk, tryptone, glucose, and glycerol (STGG) transport medium, vortexed and frozen at -70°C until used for standard microbiological culture (31). All *H. influenzae* isolated from these swabs were NTHi (30). Aliquots of maternal, cord and infant sera and breast milk were shipped on dry ice from the Menzies School of Health Research in Darwin, Northern Territory to the laboratories at the Telethon Kids Institute in Perth, Western Australia for analysis of antibody titres.

### Ear assessments

Infant’s ears were assessed by trained research nurses (and reviewed by an independent assessor) using tympanometry and otoscopy at 1-, 2- and 7-months of age and scored for presence or absence of middle ear infection (OM) in the infants’ worst ear according to recommended guidelines for clinical practice in Aboriginal and Torres Strait Islander children (30, 32). Outcomes of clinical phenotypes of OM e.g. acute OM or OM with effusion are not presented. Based on these diagnoses, sub-analyses were conducted comparing infants with OM observed at any study visit over the 7-months and those without.

### *S. pneumoniae* and *H. influenzae* antigens used in this study

*S. pneumoniae* antigens PspA1, PspA2, CbpA and Ply were prepared as previously described (33). The sequence for PD was selected based on *H. influenzae* strain 772 (34). Pure, stable and endotoxin free recombinant PD was produced by the Protein Expression Facility (The University of Queensland) according to optimised protocols and adapted from methods used previously (33). *H. influenzae* antigens OMP26, rsPilA and ChimV4 were prepared as described previously (21).

### Measurement of IgG and IgA against *S. pneumoniae* antigens PspA1, PspA2, CbpA, Ply and *H. influenzae* antigens PD, OMP26, rsPilA and ChimV4

Sera were assessed for specific IgG antibodies against *S. pneumoniae* antigens PspA1, PspA2, CbpA, Ply, and *H. influenzae* antigens PD, OMP26, rsPilA and ChimV4 using multiplex bead-based immunoassays that have been described previously (21). The final assays consisted of a 5-plex immunoassay containing PspA1, PD, ScpA (*Streptococcus pyogenes* antigen, data not shown here), OMP26 and rsPilA, and a 4-plex immunoassay containing PspA2, CbpA, Ply and ChimV4. Test sera were diluted at 1:500 in sample diluent (phosphate-buffered saline with 2% newborn bovine sera and 0.05% Tween 20 (Sigma-Aldrich)). A 3-fold dilution of standards was prepared at a starting ratio of 1:20. Diluted sera (25µL) and standards (25µL) were added to the plate and mixed with prepared beads (25µL containing 4000 beads per region) and incubated in the dark at room temperature for 30 minutes, followed by standard bioplex protocol (35).

Breast milk was assessed for antigen-specific IgA antibodies against *S. pneumoniae* antigens, PspA1, PspA2 and Ply, and *H. influenzae* specific antigens PD, OMP26, rsPilA and ChimV4 using multiplexed bead-based immunoassays as for sera. A breast milk reference sample was prepared from pooled breast milk samples from 8 donors from a maternal *S. pneumoniae* vaccination study (Princess Margaret Hospital for Children Human Ethics Committee 721EP) and diluted 2-fold starting at 1:2.5. Antigen-specific IgG titres were also measured in breast milk samples, except for rsPilA as antibody titres to this antigen in the reference sample were below the limit of detection and a standard curve could not be established for analysis. Test samples were diluted 1:20 in sample diluent and immunoassays were conducted as above for sera. Antibody titres to CbpA could not be measured in breast milk as CbpA binds to the secretory component of secretory IgA in breast milk (36).

The BioPlex^®^ 200 System (BioRad) was used to measure the fluorescence of 100 beads per specific bead region. The mean fluorescence intensity (MFI) values were generated with the Bio-plex Manager 6.0 software into arbitrary units (AU/mL). Inter-assay variability was assessed using the percentage of coefficient of variation (%CV) of MFI of the standard curve and remained within 10-15% between plates. Out of range readings were repeated at an appropriate higher or lower dilution (down to 1:20 for sera and 1:5 for breast milk) to fit within the standard curve.

### Statistical analyses

All antibody concentration data were non-parametric and therefore log-transformed to normalise prior to analysis. Cord, maternal and infant sera, and breast milk samples were then analysed using parametric paired t-tests. Unpaired t-tests were used to compare carriage and non-carriage groups, OM and no OM groups, and between 23vPPV vaccine groups. Fold changes were calculated by dividing paired cord/infant titres and maternal transfer ratios were calculated by dividing paired cord/maternal titres. A paired mixed-effects model was used to compare breast milk antigen-specific IgA and IgG tires over time. Geometric mean titres (GMT) were calculated with 95% confidence intervals (CI). All data were analysed using SPSS version 25 (IBM, Armonk, New York, United States of America) and graphs prepared with GraphPad Prism 8 (GraphPad, La Jolla, California, United States of America).

## Results

### Study cohort

**Figure 1:**
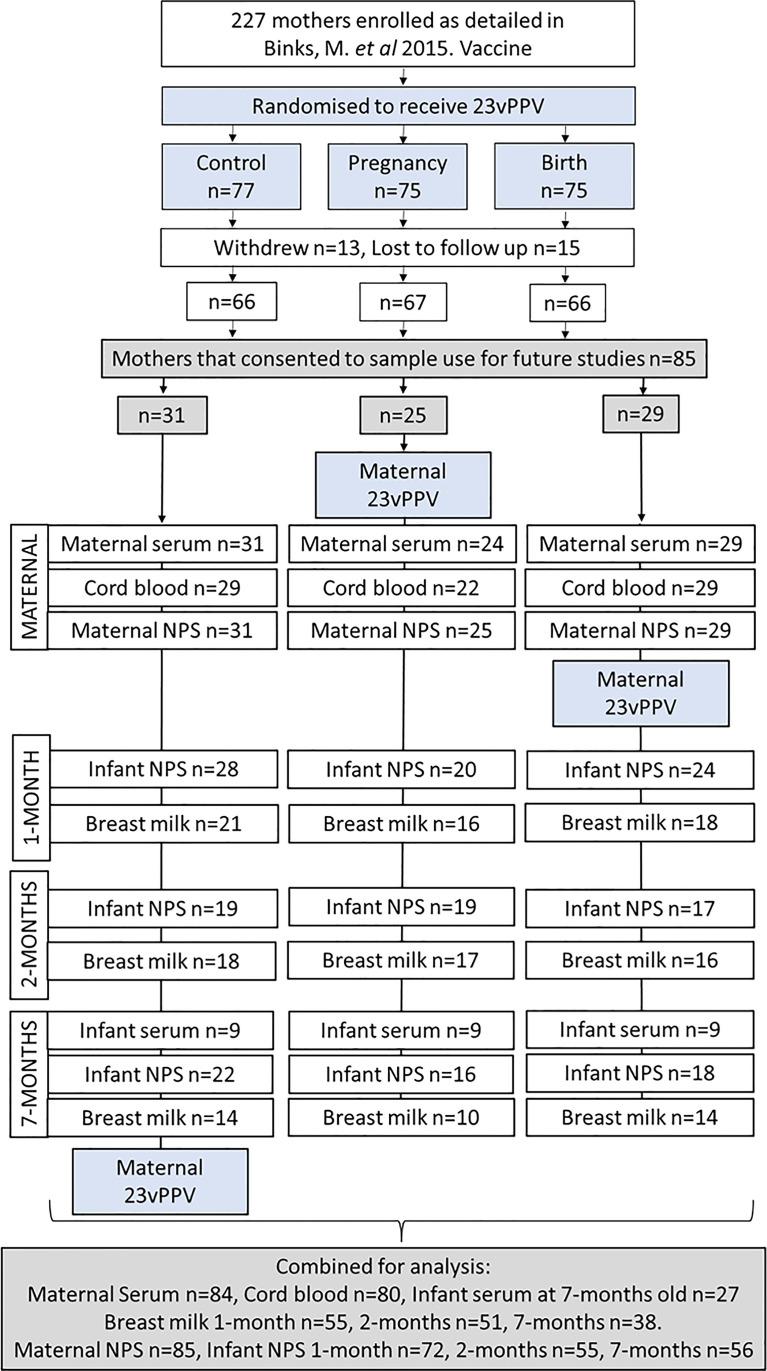
Participant flow chart and sample numbers. Of the 85 mother:infant pairs in this study 1 maternal serum sample was not collected (n=84); 5 mothers had no cord blood sera collected (n=80), and infant sera was not collected from 58 of the 85 infants (n=27).

The PneuMum study enrolled 227 mothers, of which 85 gave consent for use of their samples in future studies including this study (**Figure 1**). Of those 85 mothers, 25 (29.4%) received 23vPPV between the 30^th^-36^th^ week of pregnancy, 29 (34.0%) received 23vPPV within 4 hours of delivery and 31 (36.5%) received 23vPPV upon exit from the study (when the infant was 7-months old). The GMT of maternal, cord and infant sera IgG and breast milk IgA and IgG (**Supplementary Table S1**) for all 8 antigens studied here, were compared between the maternal vaccination groups, with no differences as expected (p≥ 0.05) (except breast milk Ply IgG at 1- and 2-months; p= 0.001). Therefore, timing of 23vPPV vaccination was not considered a confounder and groups were combined for further study analyses. Slightly more male infants were represented (56%). All babies were delivered at term, with a mean of 39.2 weeks [95% CI: 39.0 - 39.5 weeks] (**Table 1**). The average birth weight of study infants was 3452 g [95% CI: 2060 g – 4425 g] and the mean maternal age was 25.1 years [95% CI: 17 - 37 years of age] (**Table 1**).

**Table 1:** Participant characteristics as a whole and by infant OM in the first 7-months.

	Total	With Otitis Media	Without Otitis Media	P value
**Number, n**	85	44	41	–
**Maternal sera samples, n**	84	43	41	–
**Cord blood samples, n**	80	42	38	–
**Infant sera samples, n**	27	16	11	–
**Breast milk samples at 1-month, n**	55	31	24	–
**Breast milk samples at 2-months, n**	51	33	18	–
**Breast milk samples at 7-months, n**	38	29	9	–
**23vPPV in pregnancy, n (%)**	25 (29)	11 (25)	14 (34)	0.999
**23vPPV at birth, n (%)**	29 (34)	15 (34)	14 (34)	0.999
**No 23vPPV, n (%)**	31 (37)	18 (41)	13 (32)	0.815
**Male infant, n (%)**	48 (56)	25 (56)	23 (56)	0.999
**Gestation, weeks (range)**	39.24 (36-41)	39.45 (37-41)	39 (36-41)	0.071
**Maternal age at birth, years (range)**	25.10 (17-37)	24.84 (17-34)	25.38 (17-37)	0.615
**Infant birth weight, grams (range)**	3452 (2060-4425)	3443.41 (2570-4420)	3460.37 (2060-4425)	0.881
**NTHi in NPS at delivery, n (%)**	6/85 (7)	4/44 (9)	2/41 (5)	0.677
**NTHi in NPS at 1-month, n (%)**	8/72 (11)	8/36 (22)	0/36 (0)	**0.005**
**NTHi in NPS at 2-months, n (%)**	5/55 (9)	5/36 (14)	0/19 (0)	0.152
**NTHi in NPS at 7-months, n (%)**	25/56 (45)	23/39 (59)	2/17 (12)	**0.001**
**NTHi in NPS infant any time, n (%)**	28/85 (33)	26/44 (59)	2/41 (5)	**0.001**
***S. pneumoniae* in NPS at delivery, n (%)**	6/85 (7)	4/44 (9)	2/41 (5)	0.677
***S. pneumoniae* in NPS at 1-month, n (%)**	6/72 (8)	4/36 (11)	2/36 (6)	0.674
***S. pneumoniae* in NPS at 2-months, n (%)**	14/55 (26)	12/36 (33)	2/19 (11)	0.103
***S. pneumoniae* in NPS at 7-months, n (%)**	26/56 (46)	24/39 (62)	2/17 (12)	**0.001**
***S. pneumoniae* infant any time, n (%)**	35/85 (41)	29/44 (66)	6/41 (15)	**0.001**
**Breastfed at 1-month, n (%)**	65/85 (76)	34/44 (77)	31/41 (75)	0.999
**Breastfed at 2-months, n (%)**	52/85 (61)	34/44 (77)	18/41 (44)	**0.002**
**Breastfed at 7-months, n (%)**	40/85 (47)	31/44 (70)	9/41 (22)	**0.001**
**Exclusively breastfed at 1-month, n (%)**	49/65 (75)	27/34 (79)	22/34 (65)	0.566
**Exclusively breastfed at 2-months, n (%)**	35/52 (67)	25/34 (74)	10/18 (56)	0.701
**Exclusively breastfed at 7-months, n (%)**	30/40 (75)	25/31 (81)	5/9 (56)	0.190

All categorical variables analysis used a Fisher’s exact test.

Bold p values = significance of less than 0.05. Kruskal-Wallis tests for continuous variables (gestation, maternal age and infant birth weight).

Eighty-four maternal sera were collected (1 not collected), 53 cord sera (32 not collected) and 27 infant sera at 7-months old (58 not collected) (**Figure 1**). A total of 144 breast milk samples were collected. Of the 65/85 mothers who were breastfeeding at 1-month, 55 provided a breast milk sample, at 2-months, 52/85 mothers were breastfeeding with 51 providing a breast milk sample. At 7-months, 40/85 mothers were breastfeeding with 38 provided a breast milk sample. Infant study visits were scheduled at 1-, 2- and 7-months of age. There were 13 out of 85 infants who missed the 1-month study visit (no NPS collected, and no ear assessment done), 30 missed at 2-months and 29 missed at 7-months. 23 infants attended only 1 study visit, 26 infants attended 2 study visits and 36 infants attended all three study visits and had their ears assessed and NPS collected (**Figure 1**).

### By 7-months of age nearly half of the infants carried either *S. pneumoniae* or NTHi and this was associated with an OM diagnosis

Nasopharyngeal swabs were collected from all 85 mothers at the time of delivery (**Figure 1**) with maternal carriage uncommon (**Table 1**). Nasopharyngeal swabs were collected from 72 infants at 1-month, 55 infants at 2-months, and 56 infants at 7-months (**Figure 1**). At 1-month of age, 8/72 infants (11%) were colonised with NTHi, and 6/72 infants (8%) were colonised with *S. pneumoniae* (**Table 1**). At 2-months of age, 5/55 infants (9%) were carrying NTHi and 14/55 infants (26%) were colonised with *S. pneumoniae*. At 7-months of age 25/56 infants (45%) carried NTHi, and 26/56 infants (46%) carried *S. pneumoniae*. Of those infants with all 3 NPS collected, 2/36 (6%) had *S. pneumoniae* detected at all 3 study visits and 2/36 (6%) had NTHi detected at all 3 study visits, with one of those infants being positive for both pathogens at all 3 study visits.

At the 1-month study visit, 12/71 (17%) infants had OM observed, increasing to 19/53 (36%) infants at the 2-months study visit. At the 7-month study visit, 33/54 (61%) infants had OM observed. Over the three study visits, 29 (34%) children had 1 OM episode observed, 10 (12%) had 2 OM episodes observed and 5 (6%) had OM observed at all 3 visits. Over the whole 7-months of the study, 44/85 (52%) children had OM observed at 1 or more study visit.

Overall, 29/44 (66%) infants who were colonised with *S. pneumoniae* and 26/44 (59%) infants who were colonised with NTHi on at least one of their three study visits also had OM observed during at least one of their three study visits.

### Antibody titres were similar in maternal and cord sera for all antigens measured

**Figure 2:**
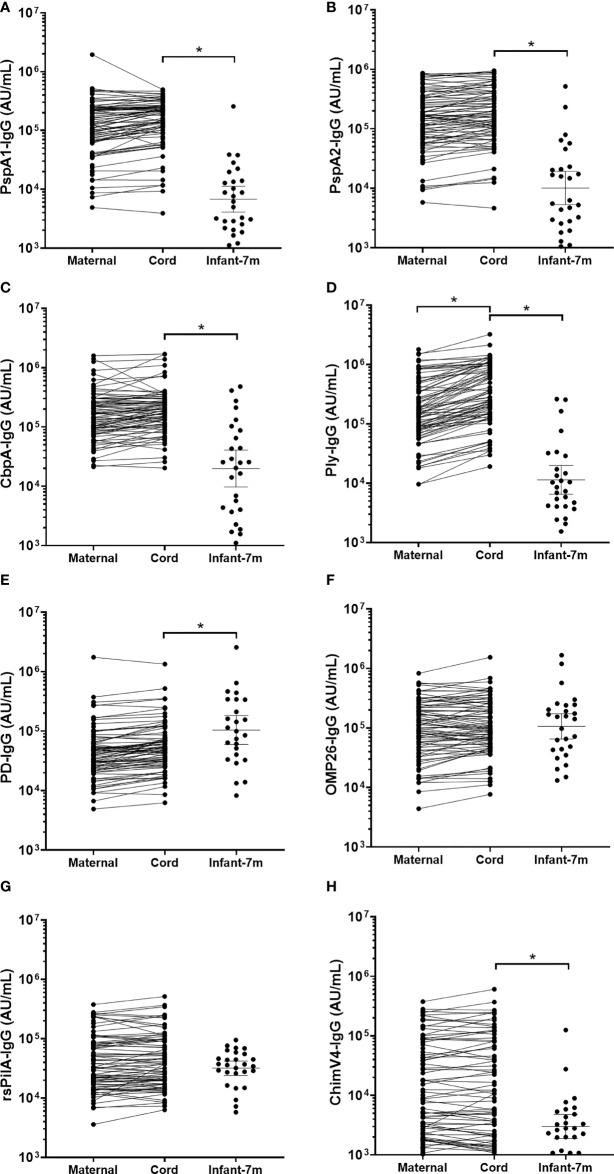
IgG titres against *Streptococcus pneumoniae* and *Haemophilus influenzae* antigens in maternal, cord and infant sera. Each point represents sera IgG titres against *S. pneumoniae* antigens **(A)** PspA1, **(B)** PspA2, **(C)** CbpA, **(D)** Ply and *H. influenzae* antigens **(E)** PD, **(F)** OMP26, **(G)** rsPilA and **(H)** ChimV4. PspA1, pneumococcal surface protein A family 1; PspA2, pneumococcal surface protein A family 2; CbpA, choline-binding protein A; Ply, pneumolysin. PD, Protein D; OMP26, outer membrane protein 26; rsPilA, recombinant soluble pilus A protein; ChimV4, chimeric vaccine antigen 4 (rsPilA and P5). * p-value <0.05, Paired t-tests were conducted on the logarithmically transformed data. All samples had measurable antibody titres.

IgG GMT in cord and maternal sera were similar for *S. pneumoniae* antigens PspA1, PspA2 and CbpA (p≥0.05; **Figures 2A–C**). For the *S. pneumoniae* antigen Ply, IgG was higher in cord sera than in maternal sera (**Figure 2D**, p=0.004). IgG titres in cord and maternal sera were similar for all *H. influenzae* antigens (p≥0.05; **Figures 2E–H**). All GMT and 95% CI are shown in **Supplementary Table S2**. The geometric mean cord/maternal IgG antibody ratios were all above 1 (anti-PspA1: 1.21 [range 1.11-1.31], anti-PspA2: 1.18 [range 1.10-1.26], anti-CbpA: 1.19 [range 1.09-1.29], anti-Ply: 1.99 [range 1.81-2.20], anti-PD: 1.27 [range 1.19-1.35], anti-ChimV4: 1.01 [range 0.93-1.10], anti-rsPilA: 1.07 [range 1.00-1.14] and anti-OMP26: 1.09 [range 1.01-1.18]).

### *S. pneumoniae*-specific IgG titres in cord sera were up to 20-fold higher than infant serum at 7-months of age

Cord blood IgG titres were higher than those in infant sera at 7-months of age for all 4 *S. pneumoniae* antigens (p<0.001). Geometric mean cord/infant IgG fold changes were as follows, 19.7-fold for anti-PspA1 [range 180 to 1.26], 14.8-fold for anti-PspA2 [range 98.1 to 0.5], 9.61-fold for anti-CbpA [range 110 to 0.25] and 21.7-fold for anti-Ply [range 216 to 0.17].

### *H. influenzae*-specific IgG titres were similar between cord and infant sera

Antigen-specific cord sera IgG GMT were lower compared to infant IgG at 7-months of age for PD (p=0.029), but higher for ChimV4 (p=0.001) (**Figures 2E, H**). There were no differences in cord versus infant IgG GMT for OMP26 (p=0.999) or rsPilA (p=0.207) (**Figures 2F, G**). All GMT and 95% CI are shown in **Supplementary Table S2**. The geometric mean cord/infant IgG fold changes 0.46-fold for anti-PD [range 10.6 to 0.02], 1.0-fold for anti-OMP26 [range 75.4 to 0.02], 4.2-fold for anti-ChimV4 [range 59.3 to 0.06] and 1.4-fold for anti-rsPilA [range 21.1 to 0.14].

### *S. pneumoniae* antigen-specific sera IgG titres were higher in infants colonised with *S. pneumoniae* in the first 7-months of life

**Figure 3:**
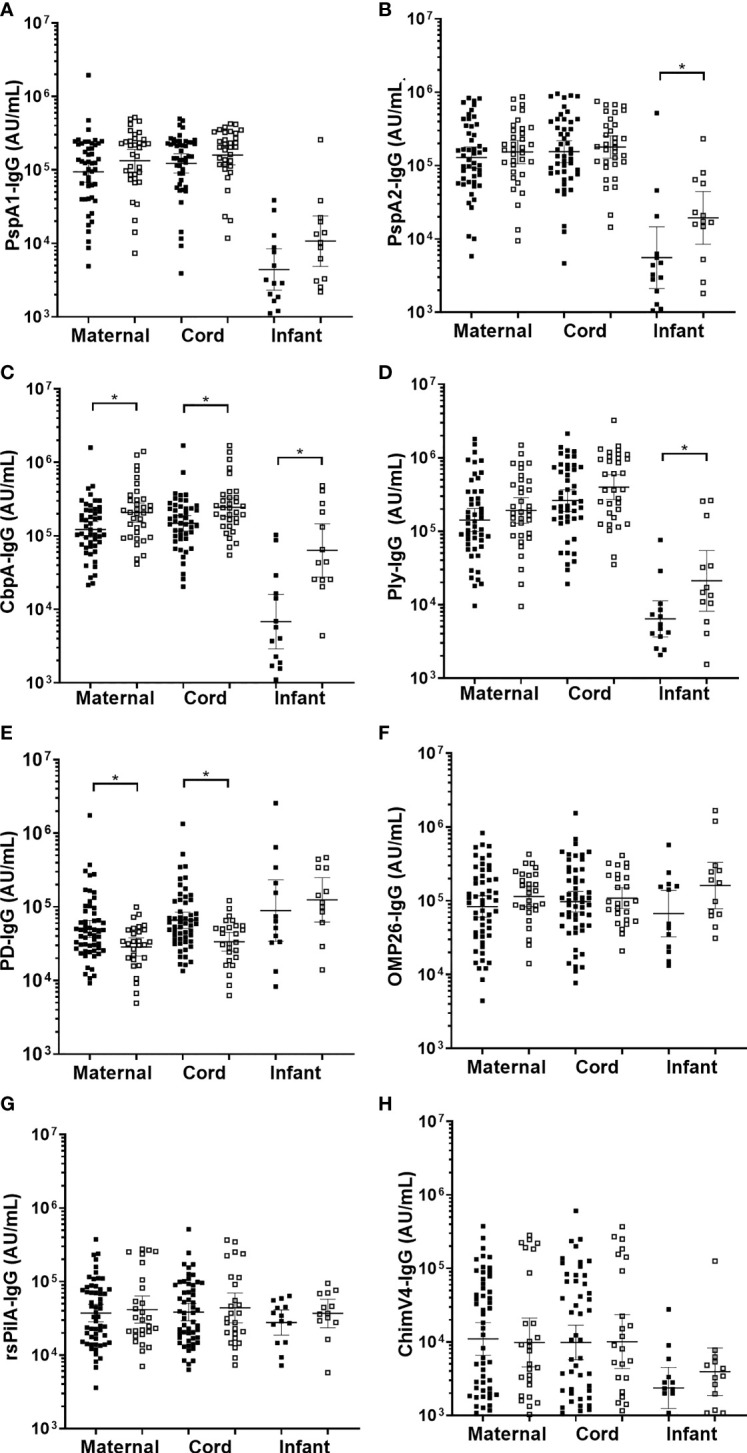
Sera IgG titres against *Streptococcus pneumoniae* or *Haemophilus influenzae* separated based on any NPS positive to *S. pneumoniae* or NTHi in the first 7-months of life, in maternal venous sera, cord sera and infant venous sera from matched mother-infant pairs. Each point represents the sera IgG titres against *S. pneumoniae* antigens **(A)** PspA1, **(B)** PspA2, **(C)** CbpA, **(D)** Ply and *H. influenzae* antigens **(E)** PD, **(F)** OMP26, **(G)** rsPilA and **(H)** ChimV4. PspA1, pneumococcal surface protein A family 1; PspA2, pneumococcal surface protein A family 2; CbpA, choline-binding protein A; Ply, pneumolysin. PD, Protein D; OMP26, outer membrane protein 26; rsPilA, recombinant soluble pilus A protein; ChimV4, chimeric vaccine antigen 4 (rsPilA and P5). *p-value <0.05, Unpaired t-tests were conducted on logarithmically transformed data. Positive NPS = open squares.

Overall, 35/85 (41%) infants had at least one NPS positive for *S. pneumoniae* over the 7-months (**Table 1**). Infant sera IgG titres at 7-months of age against *S. pneumoniae* specific PspA2 (p=0.045), CbpA (p=0.001) and Ply (p=0.025) were all higher in infants that carried *S. pneumoniae* at any study visit over the 7-months **Figure 3**). Titres of anti-CbpA IgG in cord sera (p=0.009) and CbpA IgG in maternal sera (p=0.006) were higher in those mothers whose infant had any positive NPS for *S. pneumoniae* carriage over the 7-months (**Figure 3C**).

### *H. influenzae* antigen titres were similar between infants with and without NTHi carriage, except higher anti-PD IgG titres in cord and maternal sera were associated with lower prevalence of infant NTHi carriage

Overall, 28/85 (33%) infants had at least one NPS positive for NTHi over the 7-months. There were no differences in sera IgG titres against *H. influenzae* antigens in infants carrying NTHi at any study visit over the 7-months. Infants that were not colonised with NTHi at 1-, 2- or 7-months of age had higher anti-PD IgG titres in their cord serum (p=0.002) (and their mother's serum; p=0.009) (**Figure 3E**) with no differences in OMP26, rsPilA and ChimV4 (**Figures 3F–H**).

### Antigen specific maternal, cord and infant sera IgG titres did not protect against all cause OM by 7-months of age

**Figure 4:**
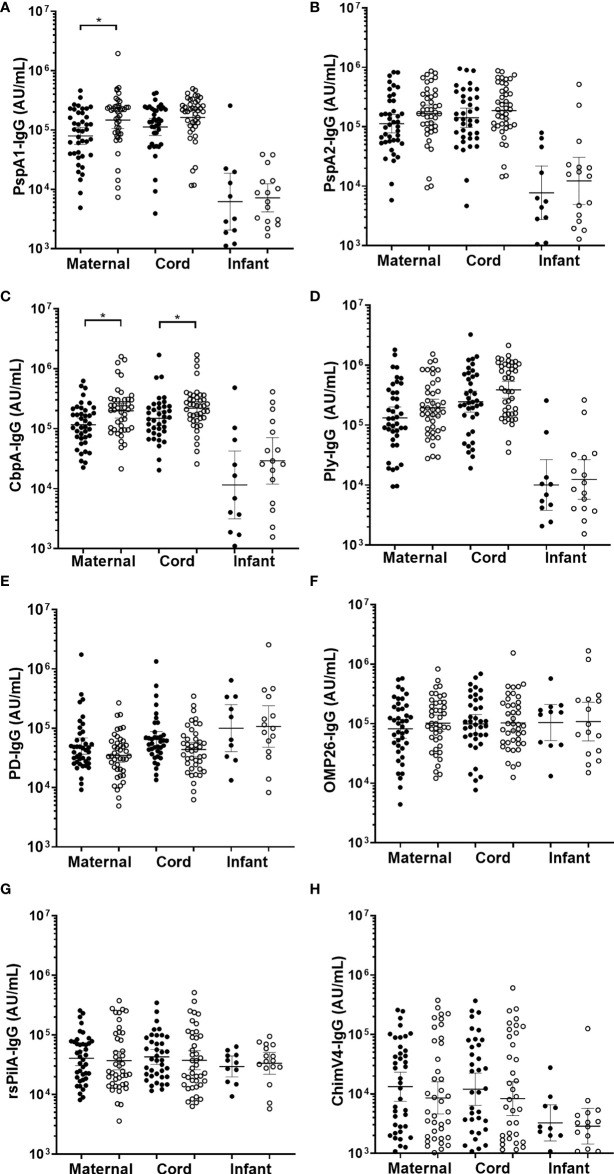
IgG antibodies against *Streptococcus pneumoniae* and *Haemophilus influenzae* antigens in maternal, cord and infant sera from matched mother-infant pairs, separated based on otitis media diagnosis in the first 7-months of life or not. Each point represents sera IgG titres against *S. pneumoniae* antigens **(A)** PspA1, **(B)** PspA2, **(C)** CbpA, **(D)** Ply and *H. influenzae* antigens **(E)** PD, **(F)** OMP26, **(G)** rsPilA and **(H)** ChimV4. PspA1, pneumococcal surface protein A family 1; PspA2, pneumococcal surface protein A family 2; CbpA, choline-binding protein A; Ply, pneumolysin. Protein D; OMP26, outer membrane protein 26; rsPilA, recombinant soluble pilus A protein; ChimV4, chimeric vaccine antigen 4 (rsPilA and P5). *p-value <0.05, Unpaired t-tests were conducted on the logarithmically transformed data. With otitis media diagnosis = open circles.

There were no differences in infant sera for GMT for anti-PspA1, anti-PspA2, anti-CbpA, or anti-Ply between those children with OM and without OM (**Figures 4A–D**). Maternal sera from mothers of infants with any OM observed by 7-months of age had higher GMT than those without OM for anti-PspA1 IgG (p=0.009) (**Figure 4A**) and CbpA (p=0.006) (**Figure 4C**). There were no differences for anti-PspA2 or anti-Ply antibody titres in maternal sera between those mothers of children with OM and without OM (**Figures 4B, D**). *H. influenzae* antigen IgG tires against PD, OMP26, rsPilA and ChimV4 in maternal, cord or infant sera were similar between children with and without an OM diagnosis (**Figures 4E–H**).

### IgA and IgG to *S. pneumoniae* and *H. influenzae* antigens were detected in breast milk, with levels sustained for 7-months post-birth

**Figure 5:**
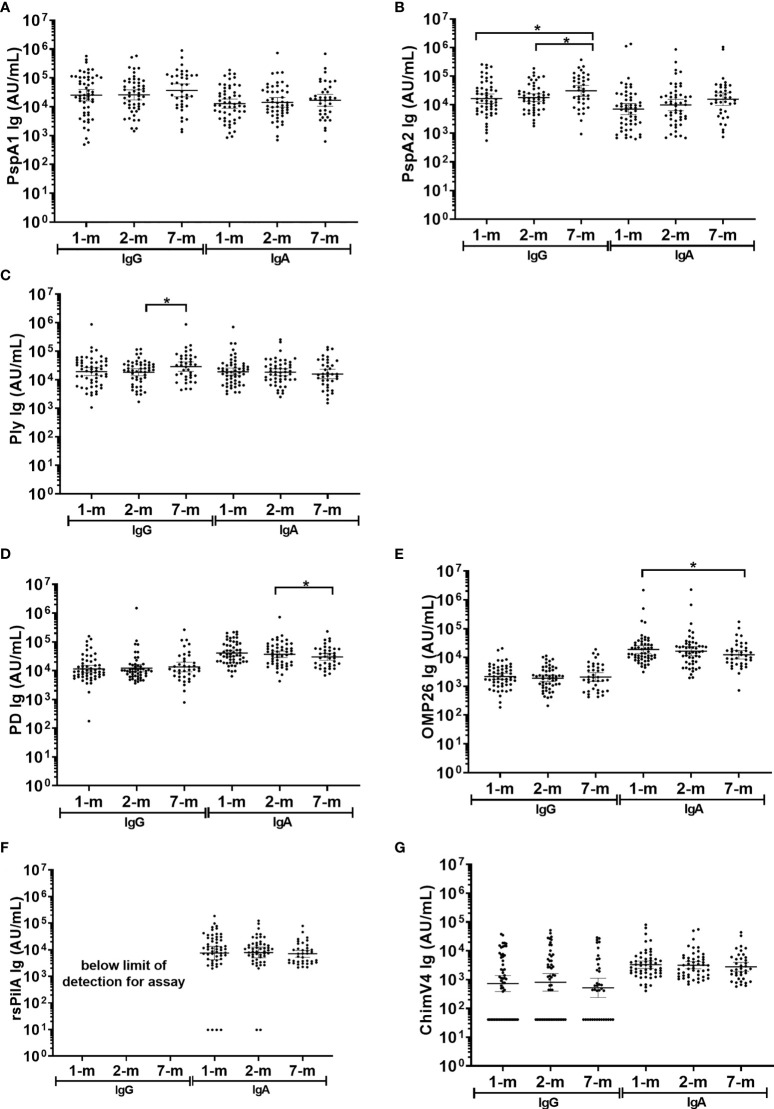
Breast milk IgG and IgA titres against *Streptococcus pneumoniae* and *Haemophilus influenzae* antigens. Each point represents the breast milk IgG and IgA titres against *S. pneumoniae* antigens **(A)** PspA1, **(B)** PspA2, **(C)** Ply and *H. influenzae* antigens **(D)** PD, **(E)** OMP26, **(F)** rsPilA and **(G)** ChimV4. PspA1, pneumococcal surface protein A family 1; PspA2, pneumococcal surface protein A family 2; Ply, pneumolysin. PD, Protein D; OMP26, outer membrane protein 26; rsPilA, recombinant soluble pilus A protein; ChimV4, chimeric vaccine antigen 4 (rsPilA and P5). * p-value <0.05, paired t-tested were conducted on the logarithmically transformed data. 1-month n=55, 2-months n=51, 7-months n=38. All samples had measurable antibody titres except for rsPilA, for which 6 samples were below the limit of detection for anti-rsPilA IgA. The assay reference breast milk had minimal rsPilA IgG therefore anti-rsPilA IgG titres could not be quantified.

In breast milk, the IgA GMT were similar at 1-, 2- and 7-months post-birth for all *S. pneumoniae* antigens tested (PspA1, PspA2 and Ply) and for 3 of the 4 *H. influenzae* antigens (PD, rsPilA and ChimV4), (**Figure 5**). The IgA GMT against OMP26 were lower in breast milk at 7-months post-birth compared to 1-month (p=0.035) (**Figure 5E**). All samples had measurable IgA titres except for rsPilA, with 6 samples below the limit of detection. All GMT and 95% CI are shown in **Supplementary Table S3**.

Fifty-one breast milk samples (35%) had no detectable anti-ChimV4 IgG and were assigned half the limit of detection (41 AU/mL). Anti-rsPilA IgG was below the detection limit in standards required for assay establishment and therefore could not be measured here, IgG to all other antigens were detectable. Anti-PspA1, anti-PD, anti-OMP26 and anti-ChimV4 IgG GMT did not change over the 7-months (p≥0.05) (**Figures 5A, D, E, G**). Anti-PspA2 IgG titres increased by 7-months of age: 1-month versus 7-months, p=0.012; 2-months versus 7-months, p=0.020, (**Supplementary Table S3** and **Figure 5B**). Anti-Ply IgG titres in breast milk also increased between 2- and 7-months of age (p=0.032) (**Figure 5C**).

### Higher anti-PspA IgG and IgA titres in breast milk were observed from mothers of infants with *S. pneumoniae* carriage, while higher anti-PD IgG titres in breast milk at 1-month were observed in infants that were not colonised with NTHi at any study visit

**Figure 6:**
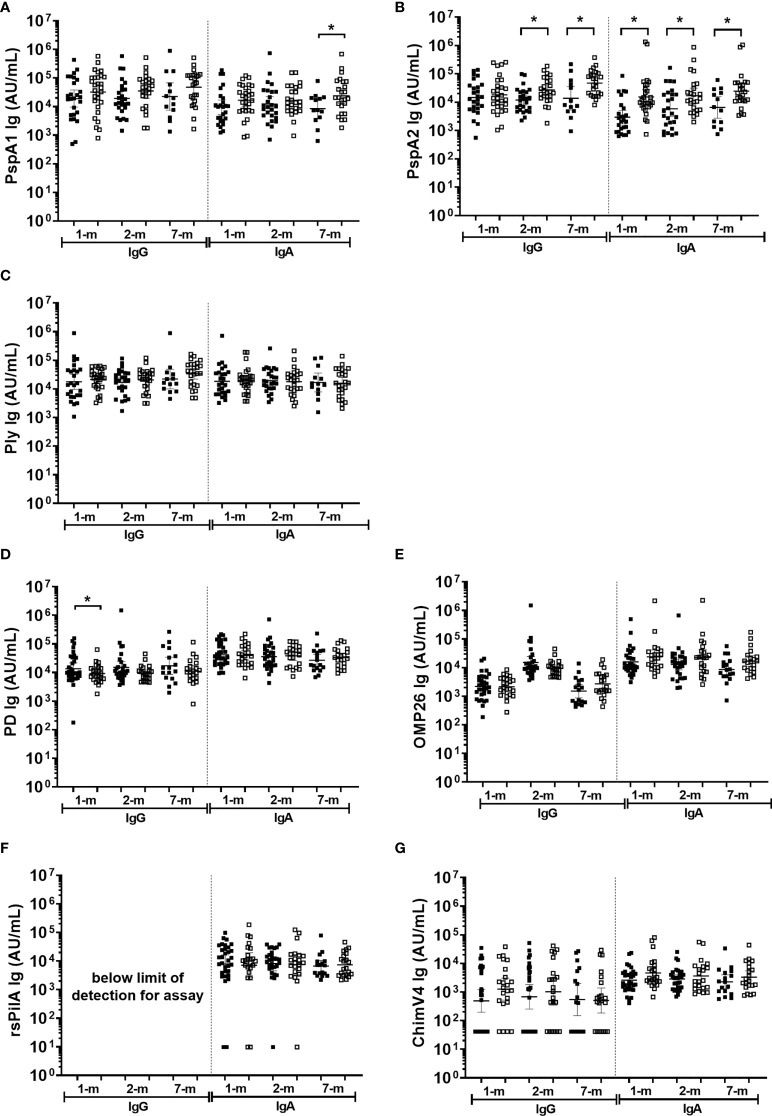
Breast milk IgG and IgA titres against *Streptococcus pneumoniae* and *Haemophilus influenzae* separated based on any NPS positive to *S. pneumoniae* or NTHi in the first 7-months of life. Each point represents the breast milk IgG or IgA titres against *S. pneumoniae* antigens **(A)** PspA1, **(B)** PspA2, **(C)** Ply and *H. influenzae* antigens **(D)** PD, **(E)** OMP26, **(F)** rsPilA, **(G)** ChimV4. PspA1, pneumococcal surface protein A family 1; PspA2, pneumococcal surface protein A family 2; Ply, pneumolysin. PD, Protein D; OMP26, outer membrane protein 26; rsPilA, recombinant soluble pilus A protein; ChimV4, chimeric vaccine antigen 4 (rsPilA and P5). Unpaired t-tests were conducted on logarithmically transformed data with * p<0.05. For the 1-month visit n=55, 2-months visit n=51, 7-months visit n=38. Positive NPS = open squares.

In general, antigen-specific IgG and IgA titres in breast milk were similar between infants that were colonised with either *S. pneumoniae* or NTHi (on at least one of the 1-, 2-, or 7-month visit) versus infants that were not colonised at a study visit (**Figure 6**). Of note was the PspA2 antibody titres in breast milk, where PspA2 IgG and IgA titres were higher in 1-month (IgA only p=0.001), 2-month (IgG p=0.006; IgA p=0.018) and 7-month (IgG p=0.006; IgA p=0.010) breast milk samples for infants colonised with *S. pneumoniae* compared with infants that were not colonised at a study visit (**Figure 6B**). Anti-PspA1 IgA titres were also higher in the 7-month breast milk sample when children were colonised with *S. pneumoniae* compared with those that were not colonised (p=0.038) (**Figure 6A**). Higher anti-PD IgG titres in breast milk at 1-month post-birth were associated with decreased prevalence of infant NTHi carriage at any study visit (p=0.041) (**Figure 6D**).

### Antigen specific antibody in breast milk did not protect against all cause OM by 7-months of age

**Figure 7:**
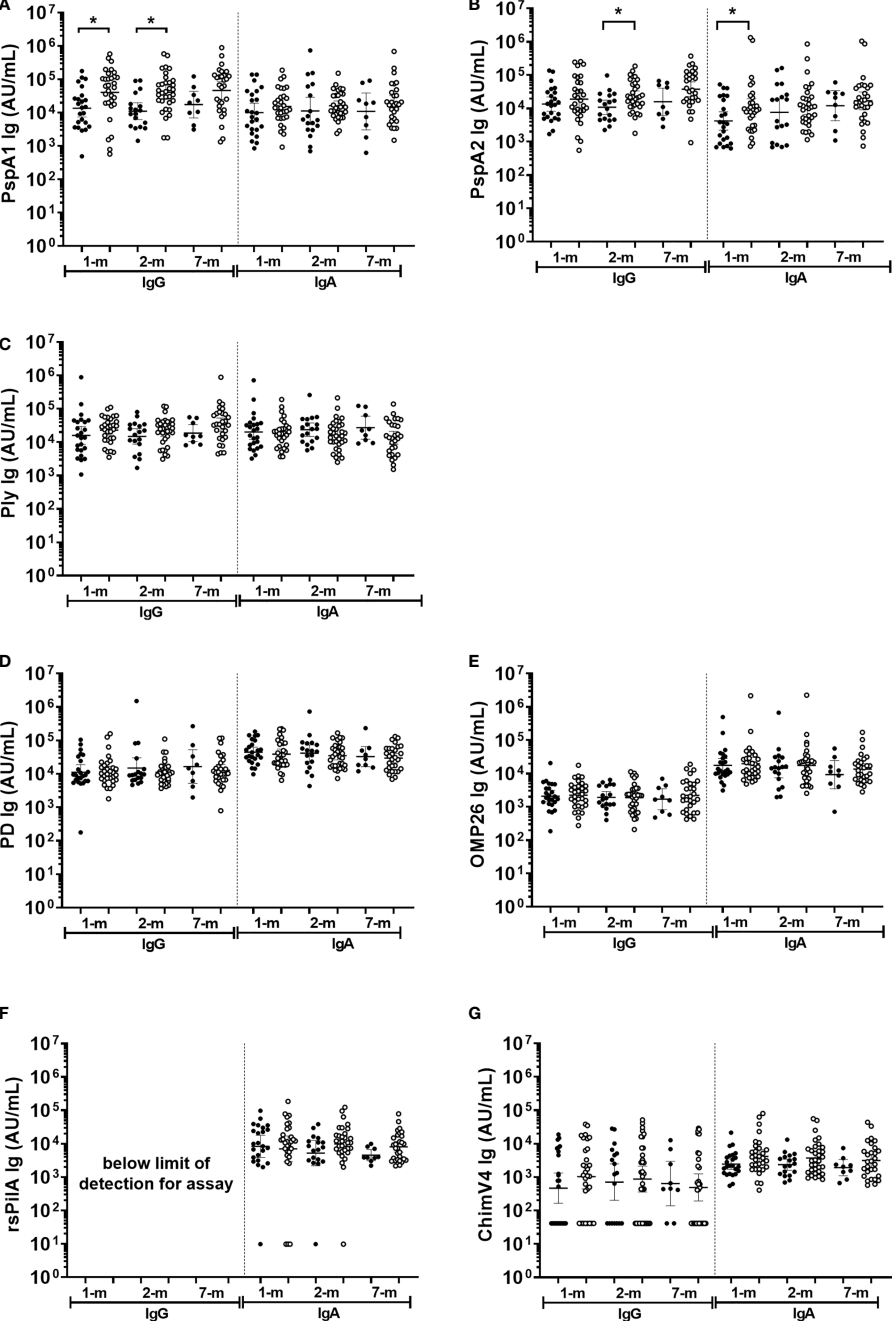
Breast milk IgG and IgA antibodies against *Streptococcus pneumoniae* and *Haemophilus influenzae* antigens separated based on otitis media diagnosis in the first 7-months of life or not. Each point represents breast milk IgG or IgA titres against *S. pneumoniae* antigens **(A)** PspA1, **(B)** PspA2, **(C)** Ply and *H. influenzae* antigens **(D)** PD, **(E)** OMP26, **(F)** rsPilA, **(G)** ChimV4. PspA1, pneumococcal surface protein A family 1; PspA2, pneumococcal surface protein A family 2; Ply, pneumolysin. PD, Protein D; OMP26, outer membrane protein 26; rsPilA, recombinant soluble pilus A protein; ChimV4, chimeric vaccine antigen 4 (rsPilA and P5). Unpaired t-tests were conducted on logarithmically transformed data with * p<0.05. 1-month n=55, 2-months n=51, 7-months n=38. With otitis media= open circles.

Antigen-specific IgA titres against *S. pneumoniae* or *H. influenzae* antigens in breast milk were similar between infants with and without an OM diagnosis at a study visit (p≥0.05; **Figure 7**). The exception was anti-PspA1 IgG and PspA2 IgA titres, where higher titres were observed in breast milk at 1-month post-birth from mothers of infants with an OM diagnosis at one or more study visit (**Figures 7A, B**) (p=0.017) and anti-PspA2 IgA GMT in 1-month breast milk sample of mother with infant OM diagnosis (p=0.048). Anti-PspA1 IgG titres were higher in the 2-month breast milk sample for mothers of infants with an OM diagnosis (p=0.001). At 2-months, anti-PspA2 IgG titres were also increased in breast milk from mothers whose infant developed OM (p=0.016).

## Discussion

Insight into maternal transfer of antibodies and early life protection against *S. pneumoniae* and *H. influenzae* is important to inform vaccine development and immunisation strategies, especially for infants at high risk of disease from these pathogens. In this study, we observed that antigen-specific natural antibody to *S. pneumoniae* and *H. influenzae* vaccine candidate antigens is present in serum and breast milk of Australian Aboriginal and Torres Strait Islander mothers. We demonstrated that cord antigen-specific serum IgG titres reflected maternal titres, indicating efficient placental transfer of antibody for every antigen tested. For the *S. pneumoniae* antibodies, we observed a 20- to 50-fold reduction in *S. pneumoniae*-specific IgG titres in infant sera at 7-months of age in comparison to cord sera titres, indicative of waning maternal antibodies in the first months of life. This has been reported in other studies of mother-infant pairs from Papua New Guinea (25), Burmese refugees at the Thai border (37), and the Philippines (23). In contrast, there was no change in *H. influenzae*-specific IgG titres between cord and infant sera at 7-months of age (i.e. no evidence of waning maternal antibodies). To our knowledge, this is the first investigation into maternal transfer of antibody to *H. influenzae* protein vaccine antigen candidates. We previously reported high titres of serum IgG against *S. pneumoniae* antigens in 1-month-old Papua New Guinean infants, which wane between 4- to 10-months of age, while *H. influenzae* antibody titres gradually increased from 1-month of age with no evidence of waning (21). This current study provides evidence that the maternal *H. influenzae* antigen-specific IgG that is available is transferred to infants (maternal:cord ratio = 1 for all *H. influenzae* antigens tested). It is therefore likely that mothers simply do not have much *H. influenzae*-specific antibody to transfer, and the infant must rely on making their own. Further support of low maternal *H. influenzae* protein antigen IgG titres available for transfer comes from a study in China, where antibody titres to *H. influenzae* PD and P6 steadily increased from 1-month of age, peaking at 7-months of age, with the lowest antibody titres observed in adults aged 21-40 years (i.e. child-bearing age) (26).

Understanding natural antibody ontogeny in mothers and infants is important when considering maternal and infant vaccination strategies. Demonstration of the ability for placental transfer of *S. pneumoniae* and *H. influenzae*-specific IgG holds promise for future immunisation strategies to confer early in life protection from these major pathogens. Our specific observation that higher *H. influenzae* PD IgG titres in maternal and cord blood were associated with protection of infants from NTHi carriage warrants further investigation. This is particularly important as this finding also applied to breast milk, where infants of mothers with higher breast milk PD IgG titres at 1-month post-birth were less likely to be colonised with NTHi by 7-month of age. This highlights the potential for maternal immunisation studies with a PD-containing vaccine (e.g. PCV10 or newer PD containing formulations in development) to delay NTHi carriage and thus *H. influenzae*-associated infections in young infants. While no correlates of protection currently exist for these antigens, placental transfer of antibodies to bacterial proteins in vaccines has been shown to be more effective than antibodies to bacterial polysaccharides in vaccines (38), further supporting the potential for maternal immunisation with PD-containing vaccines. This is of particular importance for populations such as Australian Aboriginal and Torres Strait Islanders who have high rates of NTHi carriage and NTHi OM (39, 40).

Our observation that breast milk IgA antibody titres remained constant over the first 7-months post-birth, for both *S. pneumoniae* and *H. influenzae* antigens, suggests that breast milk is a conduit for maternal antibody transfer long after birth. While breast-feeding is known to reduce the risk of *S. pneumoniae* and *H. influenzae* diseases such as OM (41, 42), the specific protective role of antigen-specific antibodies in breast milk has not been specifically investigated. A recent study using protein microarray to measure antibodies to multiple pathogens in breast milk of mothers from low- and high-income countries identified high variability in antigen-specific IgG and IgA repertoires for *S. pneumoniae* depending on geography/socioeconomic status (43). The use of breast milk to impart immunity to the surrounding environment is yet to be harnessed for prevention of *S. pneumoniae* and *H. influenzae* infections i.e. through maternal immunisation.

Limitations for this study include a single sampling point for infant sera at 7-months of age that does not enable kinetic assessment of maternal antibody waning. As middle ear samples are not able to be collected at the time of OM in Australia, the aetiology remains unknown. Some data and sample collection points were absent in this longitudinal clinical cohort, and as such the prevalence of carriage and OM could be higher than that reported. Despite these samples being collected between 2006 and 2011, serum antibody titres remain stable when samples are correctly stored and the major OM pathogens are still the same. Additionally, similar antibody waning patterns are observed in other cohorts such as the PNG study that was collected between 2014-2016 (21).

## Conclusion

Antibody to all *S. pneumoniae* and *H. influenzae* vaccine candidate antigens assessed could be measured in maternal, cord and infant sera from Aboriginal and Torres Strait Islander mother-infant pairs. The similarities between maternal and cord IgG titres confirm placental transfer of these antibodies. The absence of waning antibody to *H. influenzae* antigens between cord and infant sera supports a lack of maternal *H. influenzae* IgG antibodies available for cross-placental transfer. The observation that increased maternal anti-PD IgG (and anti-PD IgA in breast milk) may offer protection from infant NTHi carriage points to the potential value of considering PD-containing vaccines in maternal immunisation strategies. Maternal PCV10 vaccination is currently being investigated in the PneuMatters study for prevention of acute lower respiratory infection (ACTRN12618000150246). Sustained titres of antigen-specific antibody in breast milk over the first 7-months post-birth also highlights the great potential for passive-active immunisation of infants to protect against *S. pneumoniae* and *H. influenzae* disease.

## Data availability statement

The raw data supporting the conclusions of this article will be made available by the authors, without undue reservation.

## Ethics statement

The studies involving human participants were reviewed and approved by Human Research Ethics Committee of the Northern Territory Department of Health and the Menzies School of Health Research HREC and the Aboriginal Ethics Sub-Committee (HREC 2020-3800). Extensive community consultation with the PneuMum Indigenous Reference Group, Menzies Child Health Division First Nations Reference group in the Northern Territory, the Kulunga Aboriginal Unit at the Telethon Kids Institute, and the Ear Health Aboriginal Community Advisory Group in Western Australia ensured culturally appropriate processes were maintained for the additional analysis of collected specimens. Written informed consent to participate in this study was provided by the participants’ legal guardian/next of kin.

## Author contributions

ES, MB, AB, RT, PR, RA and L-AK contributed to the conceptualization of this study. KM and SC performed laboratory assays. KM analyzed the data and wrote the original draft manuscript under the supervision of L-AK. All authors have contributed to the article and have read and agreed to the published version of the article.

## Funding

This study was funded by a seed grant from the Wesfarmers Centre of Vaccines and Infectious Diseases at the Telethon Kids Institute awarded to ES, MB, RT, PR, RA and L-AK. RT is funded by a fellowship from the Passe and Williams Foundation. L-AK is funded by a fellowship from the Perron Foundation. MB is funded by Menzies School of Health fellowship.

## Acknowledgments

ChimV4 and rsPilA antigens were generously provided by Professor Lauren Bakaletz. OMP26 antigen was generously provided by Professor Allan Cripps. Ply antigen was generously provided by Professor Tim Mitchell. We also acknowledge and thank the study participants, clinical staff and the Telethon Kids Institute Kulunga team members and the Menzies Child Health Division First Nations Reference group.

## Conflict of interest

The authors declare that the research was conducted in the absence of any commercial or financial relationships that could be construed as a potential conflict of interest.

## Publisher’s note

All claims expressed in this article are solely those of the authors and do not necessarily represent those of their affiliated organizations, or those of the publisher, the editors and the reviewers. Any product that may be evaluated in this article, or claim that may be made by its manufacturer, is not guaranteed or endorsed by the publisher.
