# Supplementary material for: Evidence of maternal transfer of antigen-specific antibodies in serum and breast milk to infants at high-risk of S. pneumoniae and H. influenzae disease
Source: Front Immunol. 2022 Sep 21;13:1005344. doi: 10.3389/fimmu.2022.1005344 (PMC9535341; doi:10.3389/fimmu.2022.1005344)
Supplement: Supplementary file 1 [file Table_1.docx]

**Supplementary Table 1. Comparison of antigen-specific IgG GMT in maternal, cord and infant sera and breast milk IgA and IgG based on maternal 23vPPV vaccination status (prenatal (23vPPV given during pregnancy) or antenatal (23vPPV given at delivery or study completion).**

| **Sera** | | | | | | | | | | |
| --- | --- | --- | --- | --- | --- | --- | --- | --- | --- | --- |
|  |  | **Maternal** | | | **Cord** | | | **Infant** | | |
|  | **23vPPV** | **antenatal** | **prenatal** | ***p*** | **antenatal** | **prenatal** | ***p*** | **antenatal** | **prenatal** | ***p*** |
|  |  | **n=60** | **n=24** |  | **n=58** | **n=22** |  | **n=18 (PD;n=16)** | **n=9** |  |
|  | ***Streptococcus pneumoniae* antigens** | | | | | | | | | |
| **IgG** | **PspA1** | 115080 | 94624 | *0.462* | 140443 | 125980 | *0.659* | 7617 | 5351 | *0.509* |
|  | **PspA2** | 138229 | 136395 | *0.961* | 167148 | 156315 | *0.809* | 12806 | 6359 | *0.303* |
|  | **CbpA** | 154989 | 147605 | *0.824* | 193108 | 157362 | *0.343* | 21827 | 16596 | *0.718* |
|  | **Ply** | 172504 | 135301 | *0.410* | 344271 | 239773 | *0.209* | 11852 | 10483 | *0.837* |
|  | ***Haemophilus influenzae* antigens** | | | | | | | | | |
|  | **PD** | 38168 | 51844 | *0.180* | 47830 | 69024 | *0.111* | 133954 | 67019 | *0.228* |
|  | **rsPilA** | 34198 | 52481 | *0.089* | 36898 | 49317 | *0.270* | 34794 | 26418 | *0.355* |
|  | **ChimV4** | 9099 | 15314 | *0.263* | 9486 | 11048 | *0.764* | 2851 | 3350 | *0.745* |
|  | **OMP26** | 92598 | 89475 | *0.897* | 103276 | 94580 | *0.743* | 128410 | 73807 | *0.282* |
| **Breast milk** | | | | | | | | | | |
|  |  | **1-month** | | | **2-months** | | | **7-months** | | |
|  | **23vPPV** | **antenatal** | **prenatal** | ***p*** | **antenatal** | **prenatal** | ***p*** | **antenatal** | **prenatal** | ***p*** |
|  |  | **n=39** | **n=16** |  | **n=34** | **n=17** |  | **n=28** | **n=10** |  |
| **IgA** | ***Streptococcus pneumoniae* antigens** | | | | | | | | | |
|  | **PspA1** | 12787 | 12789 | *0.939* | 16927 | 9974 | *0.873* | 15768 | 19415 | *0.456* |
|  | **PspA2** | 8195 | 4789 | *0.330* | 13754 | 5052 | *0.309* | 15910 | 14833 | *0.315* |
|  | **Ply** | 16803 | 26611 | *0.098* | 20480 | 15257 | *0.657* | 14409 | 21515 | *0.529* |
|  | ***Haemophilus influenzae* antigens** | | | | | | | | | |
|  | **PD** | 38306 | 47730 | *0.098* | 40636 | 30381 | *0.122* | 33045 | 23368 | *0.074* |
|  | **rsPilA** | 6902 | 9372 | *0.359* | 8566 | 6642 | *0.281* | 7700 | 5532 | *0.291* |
|  | **ChimV4** | 2898 | 4508 | *0.436* | 2817 | 3961 | *0.289* | 2539 | 3621 | *0.131* |
|  | **OMP26** | 19159 | 17902 | *0.835* | 20065 | 10995 | *0.267* | 14687 | 7736 | *0.598* |
| **IgG** | ***Streptococcus pneumoniae* antigens** | | | | | | | | | |
|  | **PspA1** | 28107 | 19021 | *0.479* | 36192 | 13085 | *0.842* | 43287 | 23365 | *0.356* |
|  | **PspA2** | 18600 | 11984 | *0.418* | 23082 | 10492 | *0.720* | 36104 | 19427 | *0.631* |
|  | **Ply** | 21603 | 15346 | ***0.001*** | 20536 | 15042 | ***0.001*** | 31173 | 24251 | *0.713* |
|  | ***Haemophilus influenzae* antigens** | | | | | | | | | |
|  | **PD** | 11868 | 10615 | *0.208* | 11868 | 13158 | *0.178* | 12592 | 16369 | *0.896* |
|  | **ChimV4** | 588 | 1235 | *0.248* | 692 | 1103 | *0.751* | 450 | 795 | *0.238* |
|  | **OMP26** | 2147 | 2256 | *0.256* | 1861 | 2001 | *0.660* | 2117 | 1995 | *0.978* |

PD, Protein D; OMP26, outer membrane protein 26; rsPilA, recombinant soluble pilus A protein; ChimV4, chimeric vaccine antigen 4 (rsPilA and P5); PspA1, pneumococcal surface protein A family 1; PspA2, pneumococcal surface protein A family 2; CbpA, choline-binding protein A; Ply, non-toxic derivative of pneumolysin. *p*-values are in italics and calculated by unpaired t-test comparing log-transformed titres between groups for each sample type. Bold p values indicate a significance of less than 0.05. Total mother/infants n=85.
