# Supplementary material for: Evidence of maternal transfer of antigen-specific antibodies in serum and breast milk to infants at high-risk of S. pneumoniae and H. influenzae disease
Source: Front Immunol. 2022 Sep 21;13:1005344. doi: 10.3389/fimmu.2022.1005344 (PMC9535341; doi:10.3389/fimmu.2022.1005344)
Supplement: Supplementary file 2 [file Table_2.docx]

**Supplementary Table 2:** ***Streptococcus pneumoniae* and *Haemophilus influenzae* antigen serum IgG GMT with 95% CI for each sample type (maternal vaccine groups combined).**

|  | **Maternal** | **Cord** | **Infant** |
| --- | --- | --- | --- |
|  | **n=84** | **n=80** | **n=27 (PD;n=25)** |
| ***Streptococcus pneumoniae* antigens** | | | |
| **PspA1** | 108823 (85826-137980) | 136299 (109773-169236) | 6771 (4085-11225) |
| **PspA2** | 137706 (107994-175594) | 164079 (128559-209413) | 10141 (5314-19353) |
| **CbpA** | 152841 (125741-185782) | 182528 (150879-220817) | 19922 (9744-40730) |
| **Ply** | 160935 (123744-209305) | 311666 (241578-402088) | 11377 (6506-19894) |
| **Nontypeable *Haemophilus influenzae* antigens** | | | |
| **PD** | 41659 (33950-51118) | 52905 (43125-64905) | 104396 (59642-182730) |
| **rsPilA** | 38643 (30824-48446) | 39966 (31679-50420) | 31741 (23931-42100) |
| **ChimV4** | 10558 (6969-15997) | 9893 (6326-15470) | 3009 (1886-4800) |
| **OMP26** | 91696 (72281-116325) | 100811 (79543-127766) | 106765 (65401-174293) |

PD, Protein D; OMP26, outer membrane protein 26; rsPilA, recombinant soluble pilus A protein; ChimV4, chimeric vaccine antigen 4 (rsPilA and P5); PspA1, pneumococcal surface protein A family 1; PspA2, pneumococcal surface protein A family 2; CbpA, choline-binding protein A; Ply, non-toxic derivative of pneumolysin.
