# Supplementary material for: Evidence of maternal transfer of antigen-specific antibodies in serum and breast milk to infants at high-risk of S. pneumoniae and H. influenzae disease
Source: Front Immunol. 2022 Sep 21;13:1005344. doi: 10.3389/fimmu.2022.1005344 (PMC9535341; doi:10.3389/fimmu.2022.1005344)
Supplement: Supplementary file 3 [file Table_3.docx]

**Supplementary Table 3:** ***Streptococcus pneumoniae* and *Haemophilus influenzae* antigen breast milk IgA and IgG GMT with 95% CI for each time point (23vPPV vaccine groups combined).**

|  |  | **1-month** | **2-months** | **7-months** | ***p*** |
| --- | --- | --- | --- | --- | --- |
|  |  | **n=55** | **n=51** | **n=38** |  |
| **IgA** | ***Streptococcus pneumoniae* antigens** | | | | |
|  | **PspA1** | 12788 (8841-18497) | 14191 (9695-20773) | 16656 (10317-26889) | *0.619* |
|  | **PspA2** | 7010 (4454-11031) | 9850 (6241-15546) | 15619 (9405-25940) | *0.128* |
|  | **Ply** | 19208 (14355-25702) | 18565 (14030-24566) | 16012 (10970-23372) | *0.317* |
|  | ***Haemophilus influenzae* antigens** | | | | |
|  | **PD** | 40837 (32095-51962) | 36881 (28281-48096) | 30165 (22778-39949) | *0.102* |
|  | **rsPilA** | 7545 (4220-13489) | 7869 (4954-12500) | 7058 (5244-9499) | *0.895* |
|  | **ChimV4** | 3296 (2442-4449) | 3155 (2364-4212) | 2787 (21938-4009) | *0.306* |
|  | **OMP26** | 18784 (13762-25640) | 16419 (11359-23735) | 12407 (8821-17451) | ***0.032*** |
| **IgG** | ***Streptococcus pneumoniae* antigens** | | | | |
|  | **PspA1** | 25089 (15844-39730) | 25783 (17229-38584) | 36803 (21948-61713) | *0.082* |
|  | **PspA2** | 16367 (11179-23962) | 17748 (13064-24112) | 30671 (19732-47674) | ***0.003*** |
|  | **Ply** | 19557 (14183-26968) | 18512 (14016-24450) | 29180 (20125-42308) | ***0.016*** |
|  | ***Haemophilus influenzae* antigens** | | | | |
|  | **PD** | 11489 (8541-15454) | 12283 (9191-16417) | 13492 (9163-19868) | *0.583* |
|  | **ChimV4** | 730 (383-1390) | 808 (400-1632) | 522 (243-1123) | *0.938* |
|  | **OMP26** | 2178 (1690-2807) | 1907 (1472-2470) | 2084 (1473-2949) | *0.306* |

PD, Protein D; OMP26, outer membrane protein 26; rsPilA, recombinant soluble pilus A protein; ChimV4, chimeric vaccine antigen 4 (rsPilA and P5); PspA1, pneumococcal surface protein A family 1; PspA2, pneumococcal surface protein A family 2; CbpA, choline-binding protein A; Ply, non-toxic derivative of pneumolysin. Statistical analysis used a paired mixed-effects model on the logarithmically transformed data. Bold p values indicate a significance of less than 0.05.
